# *G. sinense* and *P. notoginseng* Extracts Improve Healthspan of Aging Flies and Provide Protection in A Huntington Disease Model

**DOI:** 10.14336/AD.2020.0714-1

**Published:** 2021-04-01

**Authors:** Serafino Teseo, Benjamin Houot, Kaiye Yang, Véronique Monnier, Guangrong Liu, Hervé Tricoire

**Affiliations:** ^1^Université de Paris, BFA, UMR 8251, CNRS, F-75013 Paris, France; ^2^School of Biological Sciences, Nanyang Technological University, Singapore; ^3^Infinitus R&D Centre, Guangzhou, China

**Keywords:** Aging, Drosophila, oxidative stress, ER stress, heart aging, *Panax notoginseng*, *Ganoderma sinense*, Huntington disease

## Abstract

In the last decades, the strong increase in the proportion of older people worldwide, and the increased prevalence of age associated degenerative diseases, have put a stronger focus on aging biology. In spite of important progresses in our understanding of the aging process, an integrative view is still lacking and there is still need for efficient anti-aging interventions that could improve healthspan, reduce incidence of age-related disease and, eventually, increase the lifespan. Interestingly, some compounds from traditional medicine have been found to possess anti-oxidative and anti-inflammatory properties, suggesting that they could play a role as anti-aging compounds, although in depth *in vivo* investigations are still scarce. In this study we used one the major aging model organisms, *Drosophila melanogaster,* to investigate the ability of four herb extracts (HEs: *Dendrobium candidum*, *Ophiopogon japonicum*, *Ganoderma sinense* and *Panax notoginseng*) widely used in traditional Chinese medicine (TCM) to slow down aging and improve healthspan of aged animals. Combining multiple approaches (stress resistance assays, lifespan and metabolic measurements, functional heart characterizations and behavioral assays), we show that these four HEs provide *in vivo* protection from various insults, albeit with significant compound-specific differences. Importantly, extracts of *P. notoginseng* and *G. sinense* increase the healthspan of aging animals, as shown by increased activity during aging and improved heart function. In addition, these two compounds also provide protection in a Drosophila model of Huntington’s disease (HD), suggesting that, besides their anti-aging properties in normal individuals, they could be also efficient in the protection against age-related diseases.

Aging has always been an important concern for mankind as shown by the large number of myths worldwide related to extended lifespan. In the last decades, the strong increase in the proportion of older people worldwide, and the increased prevalence of age associated degenerative diseases, has put a stronger focus on aging biology. The quest for an healthier long life is already mentioned two millennia ago, in the Shennong bencao jing, the first written compilation of traditional Chinese medicine (TCM)[1, 2]. In western countries aging research flied off by the 19^th^ century and was boosted by major findings at the end of the 20^th^ century[3]. Consequently, important progress has been made in our understanding of the aging process, although an integrative view is still lacking.

Aging can be considered at several levels. For many, it is directly quantitatively connected to the lifespan of the individual. However, a wider perspective consists in defining aging as a progressive reduction of fitness during the subject lifespan. A consequence of the decrease in fitness is the increased incidence of diseases in the aging individual. In this respect, slowing aging would result in increasing fitness and thus healthspan (the period of life spent in good health, without chronic diseases and aging-related disabilities), but not necessarily the lifespan of the subject. Therefore, we expect that an efficient anti-aging intervention will improve the healthspan, reduce the incidence of age-related disease and, eventually, increase the lifespan of the subject.

During the 19th and 20th centuries, the idea that aging is essentially an uncontrolled and irreversible process correlated with the metabolic activities of individuals has been the main driver of research in the field. This leading view culminated in the so called “free radical theory of aging” (FRTA), which postulates that animal species with higher metabolic rates have increased reactive oxygen species (ROS) production (mainly through endogenous production by mitochondria). This results in increased accumulation of defects in cellular components, including DNA, proteins and lipids and, consequently, in increased rate of aging [4]. Since ROS are believed to play an important role in degenerative diseases, FRTA provides a theoretical framework of the relationships between disease incidence and aging.

During the last decades, tremendous progress has been made in the genetics of aging, mainly through the use of two model organisms, *C. elegans* and *D. melanogaster* [5]. Notably, it has been shown that genetic interventions on single genes, many of them directly or indirectly related to stress protection, may significantly modulate longevity. However, in its simplest form, FRTA has been challenged by several genetic studies showing that moderate increase of oxidative stress may increase the lifespan in invertebrate and vertebrate animal models [6-8]. Thus, currently, aging is considered to be a multi-event process including loss of proteostasis, mitochondrial dysfunction, cellular senescence, stem cell exhaustion, genomic instability and epigenetic alterations. Several major genetic players involved in complex pathways may control some of these hallmarks of aging [9].

The development of anti-aging molecules has benefited from these advances. Several compounds that interfere with aging pathways, such as metformin or resveratrol, have been proposed as geroprotectors [10, 11]. In addition, the characterization of products used in traditional medicine (TM) for potential anti-aging properties is an alternative strategy directly amenable to practical applications. After functional characterization, the mode of action of anti-aging TM could be investigated by various molecular techniques. Following this line of investigation, several compounds used in TCM have been shown to possess interesting anti-oxidative and anti-inflammatory properties *in vitro* or in cellular experiments [12-14]. *In vivo* studies of these compounds have been essentially focused on therapeutic efficiency in pathological situations such as cancer or stroke [15-17]. However, only limited data from *in vivo* experiments are available regarding the efficiency of these compounds in improving healthspan during normal aging, or in slowing down age-related diseases such as neurodegenerative diseases [18, 19].

In this study, we investigated the ability to slow down aging of four herb extracts (HE) widely used in TCM (*Dendrobium candidum* extract (DCE), *Ophiopogon japonicum* root extract (OJE), *Ganoderma sinense* extract (GSE) and *Panax notoginseng* root extract (PNE)). As a model system, we used the Drosophila fruit fly, an organism well suited for *in vivo* cost-effective assays of anti-aging compounds because of its short lifespan (6 weeks), its conserved aging pathways compared to humans, and the availability of an exquisite variety of dedicated molecular genetic tools.

We show that, while these four HEs fail to extend the lifespan of experimental animals, they are able to protect them from oxidative insults. Importantly, extracts of *Panax notoginseng* and *Ganoderma sinense* also increase the healthspan of aging animals, as shown by increased activity during aging and improved heart function. In addition, animals treated with these products are more resistant to endoplasmic reticulum (ER) stress, suggesting that they benefit from improved proteostasis. In line with this finding, *Panax notoginseng* and *Ganoderma sinense* extracts provide protection in a Drosophila model of Huntington’s chorea, suggesting that they could be also efficient in the protection against age-related diseases.

## MATERIAL AND METHODS

### Raw material source and process specification of HEs Dendrobium candidum extract (DCE)

Dendrobium candidum (*Dendrobium moniliforme*) was purchased from the Kunming Institute of Botany, Chinese Academy of Sciences, and produced in Yunnan. Two kg of dry dendrobium stem were crushed, heated at 80°C in 100l of pure water and extracted for 2h. and. After filtering, 88.5L filtered extract was obtained and supplemented with 10.0% glycerin, 0.72% phenoxy-ethanol and 0.08% ethylhexylglycerin.

### Ophiopogon japonicus extract (OJE)

Ophiopogon (*Ophiopogon japonicas*) was produced in Guangdong Province and purchased from Beijing Tongrentang Group Jiren Pharmaceutical Co., Ltd. in Guangdong Province, PR China. Roots of *Ophiopogon japonicus* were grinded to 2.5kg powder and heated in distilled water (1:15 volume ratio) at 80°C for 2 hours. This step was repeated three times. Then, the extract was collected, submitted to enzymatic hydrolysis and filtered after enzyme inactivation. The final yield was 30.6L of filtered extract.

### Ganoderma sinense extract (GSE)

*Ganoderma sinense* was purchased from Tongkang Pharmaceutical Co. Ltd. in Guangdong Province, PR China, and was authenticated by Prof. Zhu-Liang Yang at Kunming Institute of Botany, Chinese Academy of Sciences. A voucher specimen (CHYX-0591) was kept at the State Key Laboratory of Photochemistry and Plant Resources in West China, Kunming Institute of Botany, Chinese Academy of Sciences, PR China. The dried powders of *G. sinense* fruiting bodies (40 kg) were extracted using refluxing 70% EtOH (3 × 130 L × 2 h) to produce a crude extract, After that, the crude extract was filtered and concentrated to 30-40% w/w under vacuum at 60°C, and then lyophilized with a freeze-dryer. Total acquired *Ganoderma sinense* extract powder was 0.72kg (yield 1.8%), which was suspended in water followed by extraction with EtOAc to afford a soluble extract (0.72 kg, 100% concentration).

### Panax notoginseng extract (PNE)

Pseudo-ginseng *(Panax notoginseng (Burk.) F.H.Chen)* was produced in Yunnan Province, and purchased from Beijing Tongrentang Group Jiren Pharmaceutical Co., Ltd. in Guangdong Province, PR China, in March 2018. 100g of powder grinded from dry pseudo-ginseng were heated in distilled water (1:20 powder:water volume ratio) for two hours (repeated three times) at 80°C and concentrated under reduced pressure to 30-40% w/w. The resulting extract was added 4 times the volume of 95% ethanol, precipitated overnight and then centrifuged for 15 minutes at 12,000 rpm before collection of the precipitate. Pseudo-ginseng polysaccharide powder (10.0g, 100% concentration) was collected under dry vacuum conditions.

### Fly strains and husbandry

Most of the experiments were performed on a *w^1118^* strain (VDRC #60200), referred below as the control strain. *foxo^D94^* flies were obtained from the Bloomington stock center (#42220). The *elav-GS* and lines used in this study have been generated in the laboratory and are already described [20, 21]. The *UAS-Htt-548-128Q* flies are a gift of F. Maschat.

Flies were cultured in a humidified, temperature-controlled incubator with a 12h on/off light cycle at 26 °C in vials containing a standard yeast-cornmeal-sucrose medium (SM: for 1l of H_2_O incorporate 60 g Springaline® inactive dried yeast, 50 g sucrose and 34 g corn flour; 6.8 g agar and 6.2 g methylbenzoate).

In all experiments, adult male flies were collected at emergence (Day 0) under light CO_2_-induced anesthesia and housed at a density of 27-32 flies per vial for subsequent experiments.

### Assays of resistance to toxic compounds or hyperoxia

Male flies were collected at emergence (Day 0) and, at Day 2, they were flipped to fresh vials containing standard medium supplemented with HEs. At Day 4, they were flipped to fresh vials containing test medium supplemented with HEs. The survival of the flies was assessed twice a day until all individuals died. For hyperoxia resistance assay, a similar scheme was followed before flies being transferred inside a tank with >95% O_2_ atmosphere that was flushed every day.

Test media contained 1.3% low melting agarose in water, 1% sucrose, and either 5 mM Paraquat (an inhibitor of mitochondrial complex I), 1% (v/v) H_2_O_2_ or 3 µM of tunicamycin (an inducer of endoplasmic reticulum ER stress) according to the toxic compound assayed in each experiment. Compounds were incorporated in the media at 45°C. For experiments using *foxo^-^* flies, Paraquat concentration was lowered to 2.5 mM according to the higher sensitivity of these flies to oxidative stress.

### Survival experiments

For longevity experiments, male flies were collected 0-12 h after emergence under light CO_2_-induced anesthesia and housed at an initial density of 27-32 flies per vial. They were flipped to fresh vials and scored for death every 2-3 days throughout adult life. SM medium supplemented or not with HEs were used for these survival experiments. In one case, we also assayed a poorer medium (PM: for 1l of H_2_O incorporate 20g Springaline® inactive dried yeast, 50 g sucrose and 8 g corn flour; 6.8 g agar and 6.2 g methylbenzoate).

### Smurf assay

In previous studies we have shown that gut permeability undergoes a sudden increase 3 days prior to a fly’s death [22, 23]. This change is easily perceivable as blue coloration appears across the whole body of flies fed on medium including a blue dye (Smurf phenotype) [22]. In order to investigate HEs effects on such a phenomenon, flies were aged on SM medium supplemented or not with HEs until the day of the Smurf assay (39 days after emergence), in which dyed medium was prepared using standard medium with blue dye #1 added at a concentration of 2.5% (w/v). Flies were kept on dyed medium overnight. A fly was counted as a Smurf when dye coloration could be observed outside of the digestive tract.

### Assays of locomotor activity

Male flies were assayed at appropriate intervals in Trikinetics devices, with a 1 mn integration window, for at least 3 days. Medium in the activity tubes was composed of 5% sucrose and 1.35% agar complemented or not with the HEs at the desired concentration. Analysis of the data was performed with ShinyR-DAM software [24].

### In vivo imaging of fly hearts and movie analysis

Flies expressing a mitochondria tagged GFP protein into the cardiac tube were anesthetized with FlyNAP (Carolina Biological Supply Company). The anterior part of the heart (abdominal segments A1/A2) was observed with a Zeiss Stereo Lumar.V12 Stereomicroscope, with a NeoLumar S 1.5× objective. Video movies were acquired with an Hamamastu Orca Flash 4.0 LT camera (50 frames per second, 501 frames per movie). Between 21 and 55 flies were imaged per condition. To analyze the movies and extract the heart parameters, we followed the analysis method described in [25].

### Calorimetry

Flies were analyzed by indirect calorimetry using the Calofly, a device allowing to measure simultaneously metabolic activity of fly groups or single individuals (Sable Systems Europe GmbH). Briefly, it is constituted of 32 animal respirometer chambers connected to flow devices for generation of main flow, subsampling and flushing, and a gas flow shifter that distributes the flow in a Li-7000 CO_2_ analyzer (Li-COR biosciences) and an Oxzilla O_2_ analyzer (Sable systems). Dedicated software treats the data and extracts values of O_2_ consumption and CO_2_ production. The device was used in a stop flow mode with individual flies in respirometer chambers and a 2h cycle of analysis. Values presented in this study correspond to two days of data collection.

### Statistical analysis

Statistical analysis was performed with the GraphPad Prism 6 software. For most experiments, statistical significance of the different conditions was assessed with non-parametric Anova (Kruskal-Wallis test with Dunn's post hoc test) and results with p<0.05 were considered as significant. For longevity curves analysis, significance was assessed using the LogRank test.

## RESULTS

### TCM HEs do not exhibit significant toxicity and do not affect feeding behavior in Drosophila

We first wanted to test whether HEs exhibits some toxicity or induce changes in the flies’ feeding behavior, which could affect the outcome of the experiments (e.g., flies eating less or more than in normal conditions, which may affect the action of the HEs as well as survival and life history traits). Therefore, we first allowed female flies to lay eggs on medium supplemented by 1% (V/V) of HEs. The survival from eggs to adult flies were scored and compared to survival on control (unsupplemented) medium. We did not observe significant changes in survival for any of the HEs (Supplementary Fig. 1A). In a second step, adult flies reared on similar media were then tested for feeding behavior by measuring, after a short feeding period (1h), their internal concentration of a blue dye incorporated in the food. Dye concentration was used as a proxy for quantifying food consumption. Using this assay, we did not detect significant differences between the 4 HEs compared to the control (Supplementary Fig. 1B). We concluded that the 4 HEs were suitable for subsequent experiments.

### HEs provides in vivo resistance to Paraquat-induced oxidative stress in adult Drosophila

Then, we asked whether HEs ingestion provides *in vivo* protection against oxidative stress in adult male flies. We first focused on Paraquat, an inhibitor of mitochondrial complex I that leads to increased production of superoxide ions. Increased resistance to this stressor has been widely documented to be correlated to increased lifespan in different genetic contexts [26, 27]. Flies were pre-treated with the HEs for 2 days before being transferred to a Paraquat-containing medium in presence of HEs and scored for survival.

**Figure 1.**
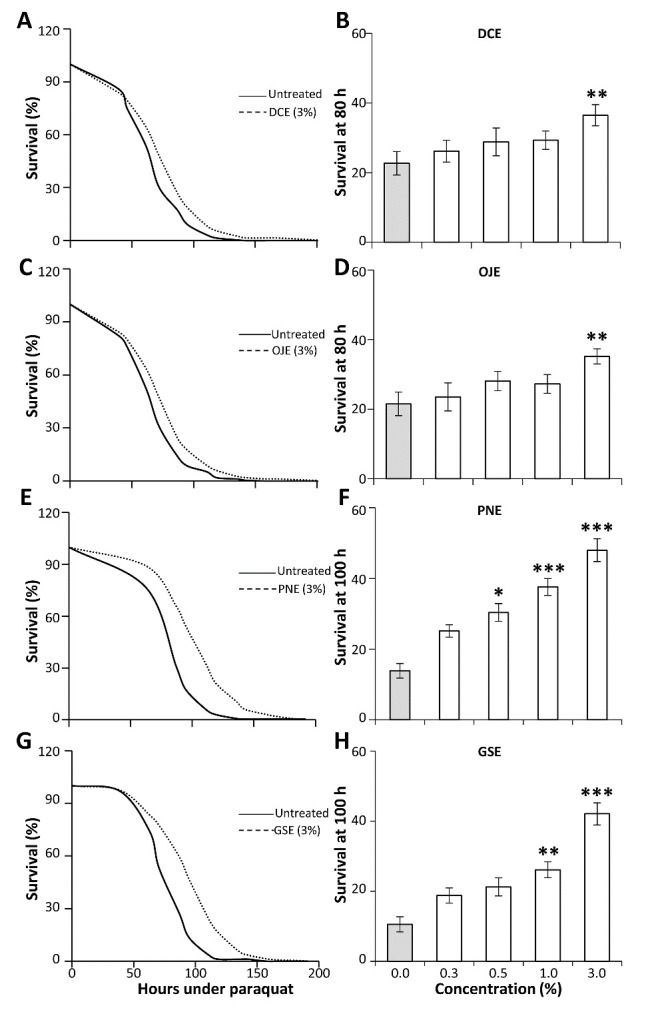
HEs prevents Paraquat induced toxicity in adult flies. 4-day old flies raised on feeding media treated with DCE (A, B), OJE (C, D), PNE (E, F) and GSE (G, H) at different concentrations were challenged with 5 mM Paraquat-induced oxidative stress. Left panel: Survival curves of flies are plotted for the maximal concentration assayed (dotted curves) and compared to control flies (plain curves). Right panel: Percentage of surviving flies at 80 h (A, C) or 100 h (E, G) are plotted for the different concentrations of HEs assayed. All HEs protected the flies against Paraquat-induced stress in a concentration-dependent manner, PNE and GSE being the most potent compounds (E-H). Error bars: SEM, number of tubes containing 30 flies: N_tubes_=9. Significant differences with untreated controls are shown *: p<0.05; **: p<0.01; ***: p<0.001.

Mild protection against Paraquat can be observed for DCE and OJE, especially at the highest concentration of 3% (V/V) where mean lifespans were increased by 12% and 13% respectively (p<0.001; Fig. 1A-D). GSE and PNE are more potent and provide a strong *in vivo* concentration-dependent protection against Paraquat-induced oxidative stress (p<0.001; Fig. 1E-H). At 3% concentration, fly mean lifespans were increased by 32% and 24% respectively. These results were confirmed in a second independent experiment.

**Figure 2.**
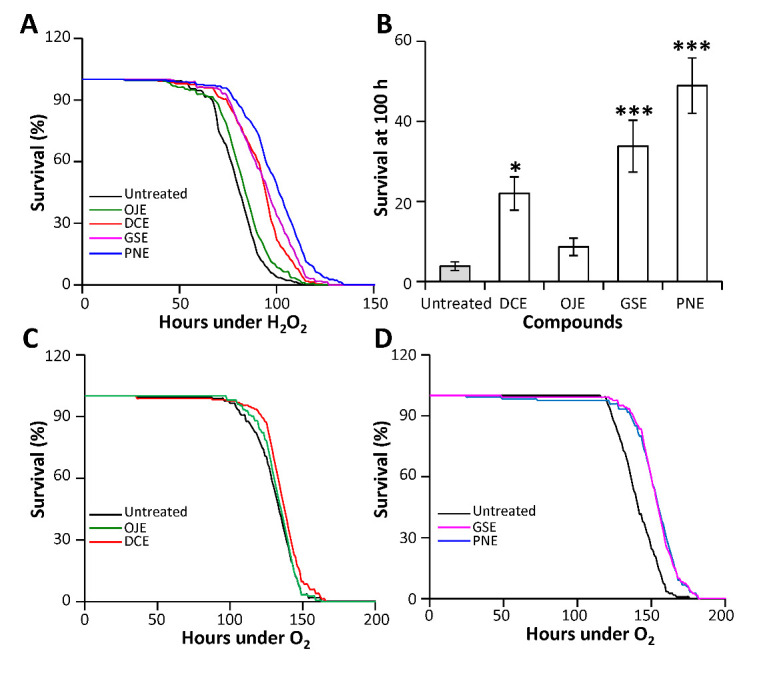
HEs provide protection against various oxidative stress in adult flies. 4-day old flies, untreated or treated with 3% HEs into the feeding medium, were challenged with 1% H_2_O_2_ (A, B) or 90-95% O_2_ atmosphere (C, D). A) Survival curves on 1% H_2_O_2_ medium emphasizing protection by all HEs except for OJE. B) After 100h on H_2_0_2_ medium, flies treated with OJE did not show significant increase in survival compared with control flies, while DCE, PNE and GSE treatments provided significant protection against H_2_O_2_-induced oxidative stress. C-D) In hyperoxic conditions neither DCE nor OJE protected significantly the flies (median lifespan change <3%) (C), while PNE and GSE treatment still improve their survival with respectively 10% and 9.3% increase in median lifespan (Logrank pvalue<0.0001) (D). Error bars: SEM, number of tubes containing 30 flies: N_tubes_ ≥ 7. Significant differences with untreated controls are shown: **: p<0.01; ***: p<0.001. Values for Logrank (Mantel-Cox) test of curves in A) are 0.03 for OJE and <0.0001 for all the other conditions when compared to the control curve.

### HEs provides differential in vivo resistance to a broad panel of oxidative stressors in adult Drosophila

Oxidative stress is a generic denomination that covers many different processes mediated by external or endogenous oxidants or free radical generating compounds. Specificity of molecular responses and protection against different oxidative stressors have been observed in molecular studies and genetic screens in yeast and drosophila [28-30]. Therefore, to extend our previous results, we investigated whether HEs modulate the *in vivo* sensitivity to different stressors.

In a first experiment, we fed the flies with medium supplemented with 3 % hydrogen peroxide (H_2_O_2_) and followed their survival. DCE, GSE and PNE provided to the flies a significant *in vivo* protection against H_2_O_2_ induced oxidative stress with increases in mean lifespan of 16, 17 and 23% respectively (p<0.001; Fig. 2A and B). In contrast, OJE was not able to provide significant protection against hydrogen peroxide induced stress (change in mean lifespan= 2%; p=0.03).

**Figure 3.**
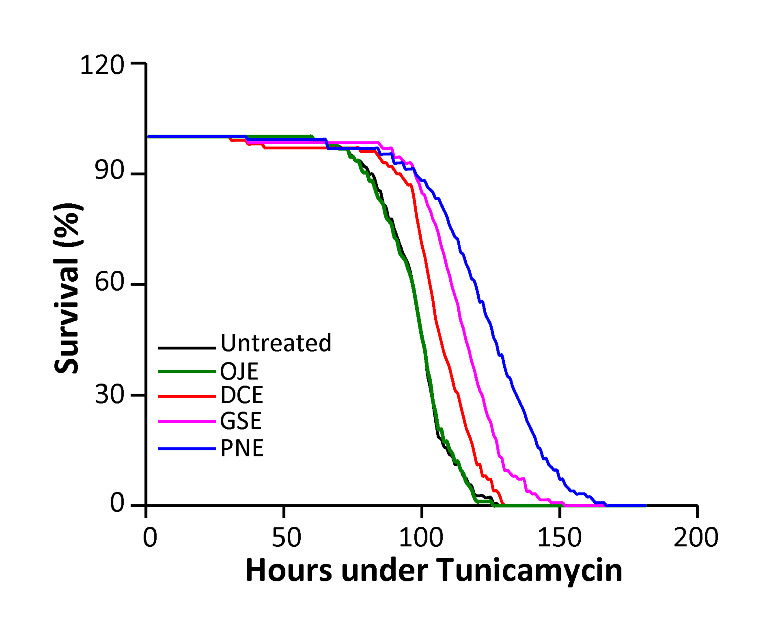
PNE and GSE protects against ER stress. Flies were treated with 3% HEs media and their sensitivity to ER stress induced by 3 µM tunicamycin was assayed. All experimental flies, except for those treated with OJE (p=0.9), exhibit a significant resistance to ER stress compared to untreated control flies (p<0.0001).

In a second experiment, we submitted flies to an hyperoxic stress (a 90%-95% O_2_ atmosphere) and analyzed their survival when treated with a concentration of 3% HEs. Compared to control (untreated) flies, we did not find significant protective effect of DCE and OJE with changes in median lifespan lower than 2.6% (Fig. 2C). In contrast, GSE and PNE treatments provided a significant *in vivo* protection against hyperoxic stress, increasing median lifespan by 10% and 9.3% respectively (p<0.0001; Fig. 2D).

In summary, all tested HEs may provide *in vivo* protection against oxidative stress. However, our experiments uncovered stress specific potencies. PNE and GSE were the most potent compounds being able to protect flies against all the oxidative stresses tested in this study. DCE protects flies against Paraquat- and H_2_O_2_-induced stresses but had no significant effects against hyperoxic stress. OJE has only a mild protective effect when the flies are challenged with Paraquat. These findings point out the need to assay anti-oxidative properties of TCM compounds in multiple *in vivo* assays to clarify their potencies.

**Figure 4.**
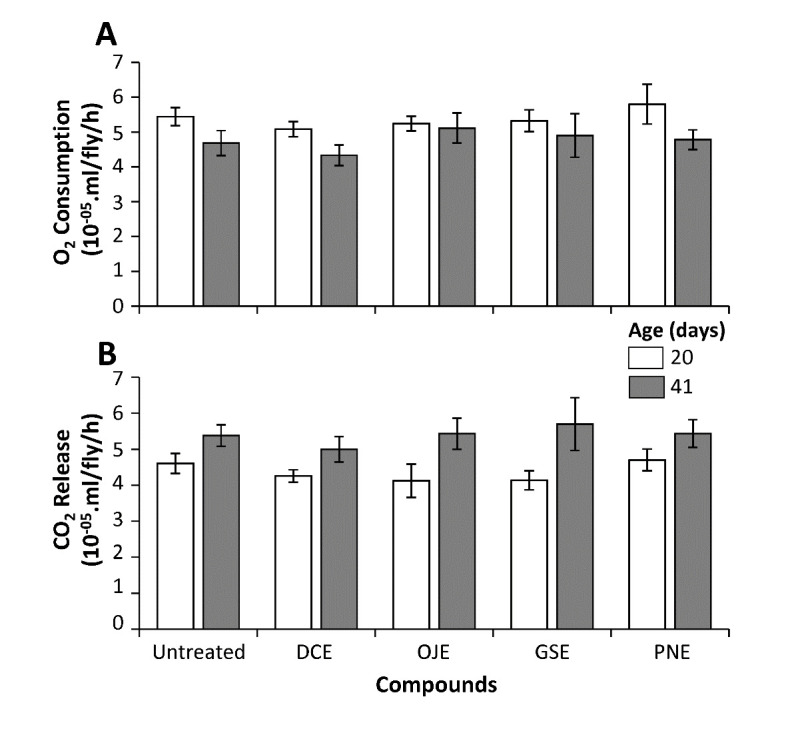
HEs treatments do not modify energetic metabolism. Oxygen consumption (A) and CO_2_ (B) release in middle-aged (20 days, light bars) and aged (40 days, dark bars) individual flies treated with HEs or untreated. No significant differences were observed between treated and untreated animals at both ages. N_flies_>=5.

### GSE and PNE protect against endoplasmic reticulum (ER) stress

Until present, the effects of traditional medicine compounds have been primarily investigated for their anti-oxidative properties. We asked whether such compounds could also provide protection against other stresses involved in aging and diseases, and notably ER stress. We therefore assayed the survival of flies treated with HEs and challenged with tunicamycin, a well-known inducer of ER stress. We observed that flies treated with OJE show similar sensitivity to tunicamycin-induced ER stress than control flies (p=0.9; Fig. 3). In contrast, flies treated with DCE, GSE and PNE exhibited a strong increase in resistance to tunicamycin induced ER stress (p<0.0001; Fig. 3). Once again, GSE and PNE were the most potent compounds, increasing median lifespan of flies by 16% and 23% respectively, compared to 7% for DCE. To our knowledge, this is the first report of an *in vivo* protective effect of GSE and PNE on ER stress.

**Figure 5.**
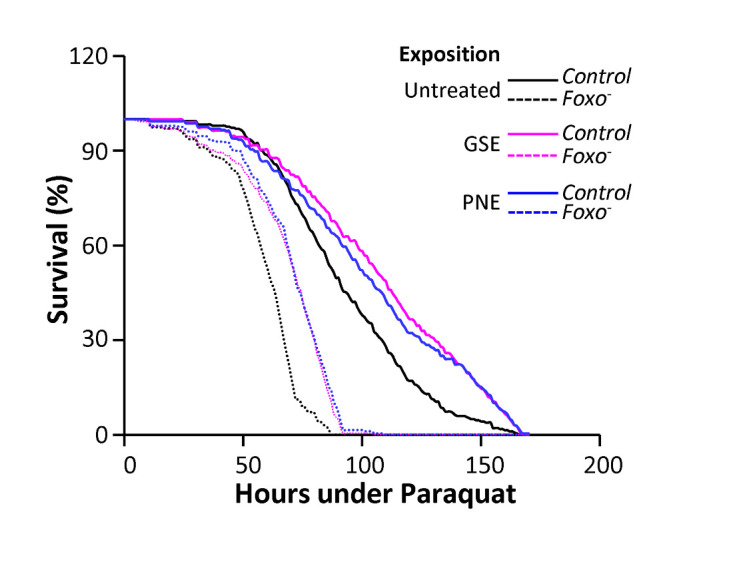
GSE and PNE protect Foxo-deficient flies against Paraquat. Control flies (full lines) and Foxo-deficient flies (dotted. lines) were challenged with 2 mM Paraquat in presence of 3% GSE or PNE and compared to untreated flies. Survival curves show that the *foxo*- mutant flies are significantly more sensitive to Paraquat than control flies (p<0.0001). In the absence of Foxo, GSE and PNE treatments are still able to significantly protect the flies against Paraquat, increasing mean lifespan by 16% and 19% respectively (p<0.0001).

### HEs treatment does not modify fly respiratory function

Considering the strong interaction between metabolism and stress responses, we asked whether HEs treatment induced changes in energy metabolism. We measured oxygen consumption and CO_2_ release in middle age (20 days) and aged (40 days) animals treated with HEs and compared their values to those obtained for untreated animals. A 32-chamber respiratory device (CaloFly, Fig S2a), working in a stop flow mode, allowed us to measure simultaneously treated and control flies in the same experiment, avoiding spurious fluctuations. At each time point, we were unable to detect significant changes between treated and control flies, neither for oxygen consumption nor CO_2_ release (Fig. 4). Interestingly, all the fly groups presented a significant drop in respiratory coefficient between 20 days and 40 days (Supplementary Fig. 2B), as observed during human aging [31], suggesting that this aging trait is not modified by HEs treatment.

### GSE and PNE protect flies against Paraquat-induced stress in a Foxo-independent manner

*foxo* is a stress protective master gene which is repressed by the IGF1/insulin pathway and has been associated to lifespan extension in many species, from worms to rodents (reviewed in [5]). Flies with genomes containing a single *Foxo* gene as well Foxo-deficient flies are viable [32]. We asked whether activation of Foxo could mediate the antioxidative protection of GSE or PNE, by investigating the survival of *foxo^-^* animals. We found that these animals are more sensitive to Paraquat than control flies (Fig. 5). However, GSE or PNE treatments still provide protection against Paraquat in these animals, to the same extent as in control flies (Fig. 5). Therefore, Foxo is dispensable for the GSE- or PNE-induced protection of flies against Paraquat induced stress, which is mediated by another pathway(s).

### HEs treatment does not increase fly lifespan

We concluded from previous experiments that at least some HEs protect the organism against a large panel of aging related stresses. Thus, we investigated whether such protection could impact the lifespan of the flies. We collected male flies at emergence and, by day 3, flipped these animals every 2 days on media containing HEs at a concentration of 3%.

**Figure 6.**
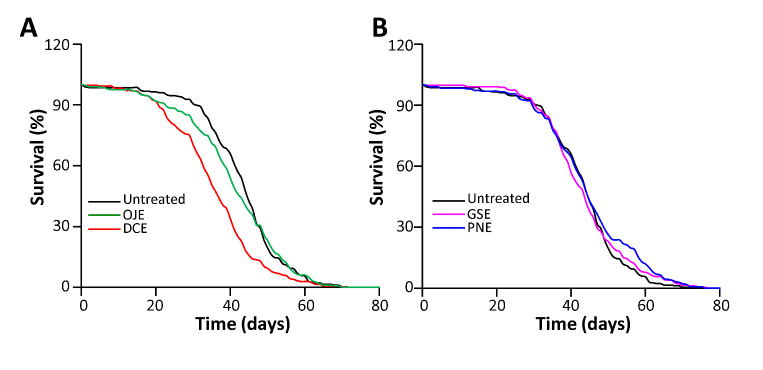
HEs treatment do not increase fly lifespan. Flies were treated with 3% HEs media along their lifespan and survival curves were scored. (A) DCE-treated flies exhibit decreased median survival (-18.2%; p<0.0001). A slight decrease in survival (-6%) was observed with OJE treatment but the survival curve does not significantly differ from the control (Logrank analysis, p=0.4). (B) PNE and GSE treatment do not have a significant effect on lifespan (p>0.01). Number of flies analyzed: N_flies_> 270 for each condition.

Unexpectedly, we found that none of the HEs increase fly lifespan. In the case of treatment with DCE, mean lifespan was significantly reduced (-17%, p<0.0001), indicating potential toxicity of long-term treatment with this compound in Drosophila (Fig. 6A). Mean lifespan of OJE-treated flies was slightly lower than the one of control flies (-5%), but the survival curves were not found to differ from the one of control flies (LogRank analysis, p=0.4). Survival curves and median lifespan of GSE- and PNE-treated flies do not differ significantly from the control (Fig. 6B). Accordingly, no significant differences of intestinal permeability, an indicator of imminent death [22], were observed in old flies (Supplementary Fig. 3). Thus, while HEs treatment has protective actions against short term stress insults in Drosophila, this increased protection does not transduce in lifespan extension after continuous treatment.

Subsequently, we investigated whether such interventions may however improve the healthspan of aging animals. For these experiments, we focused on PNE and GSE, which are the most potent protective molecules against oxidative and ER stress in previous experiments and do not exhibit toxicity on long term treatment in fly.

### GSE delays the decline of locomotor activity in aging individuals

**Figure 7.**
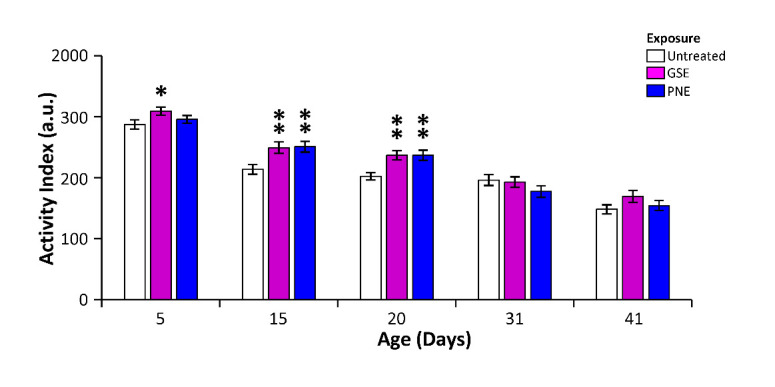
GSE and PNE delay the decline of locomotor activity in aging flies. Flies were treated with 3% of PNE or GSE media along their lifespan and their daily total activities were recorded by Trikinetics activity monitors at different time points. Aging lead to a steady decline in total daily activity from 5 to 40 days in control untreated flies (white bars). At intermediate ages, both GSE (purple bars) or PNE (blue bars) treatments significantly increased the total activity in 15- and 20-day old flies. However, these treatments had no effect on locomotor activity decline at further time points (31 and 41 days). Error bars: SEM, N_flies_>120. *: p<0.05; **: p<0.01.

One hallmark of aging in many species, including flies, is a decline of locomotor activity. In control untreated flies, one observes a steady decline in total daily activity from 5 days to 40 days (Fig. 7). According to previous results, we asked whether treatments along their lifespan with 3% of GSE or PNE, which provide multi-stress protection without changing lifespan, could counteract this decline.

PNE treatment has no significant effect on aging-related locomotor activity of young flies (5 day old), while we observed a mild increase (8%, p=0.014) with GSE treatment. At latter, aging control flies experience a strong decrease in total activity. Interestingly, both GSE and PNE treatments significantly increase the total activity in 15- and 20-day old flies (+17%, p<0.006, Fig. 7). However, activity did not change in older flies (40 day old). Thus, GSE or PNE slow down the locomotor activity decline during aging but are unable to prevent the final drop preceding death. From these results, we concluded that GSE and PNE have a limited but positive effect on fly healthspan during the course of aging.

### DCE and GSE improves cardiac function during aging

Another common feature of aging in various species, including flies and humans, is a progressive decline of cardiac function. We have previously characterized this decline in flies, showing evidence for an increase of the cardiac period during aging [25]. Since this feature can be partially reversed by decreasing oxidative stress by genetic or pharmacological means, we investigated whether DCE, OJE, GSE or PNE treatment may improve these aging features.

**Figure 8.**
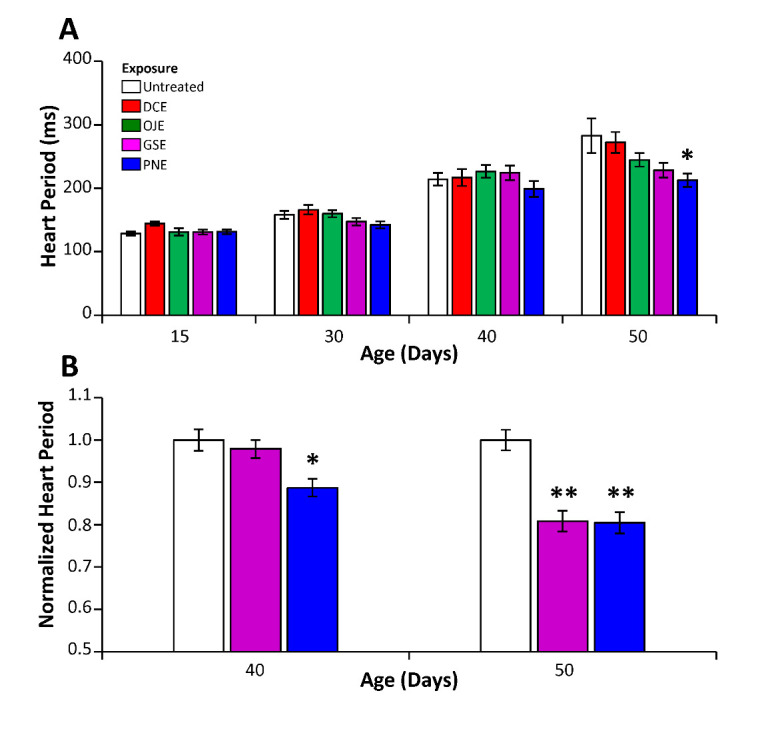
GSE and PNE improve cardiac function during aging. (A) During aging, the cardiac period of control untreated flies (white bars) exhibits a steady increase, doubling between 15-day and 50-day old flies. An initial experiment showed that DCE and OJE treatment (red and green bars) did not impact this age-related evolution significantly. By contrast, in the oldest flies (50 days), PNE treatment (blue bars) rescued significantly this phenotype (p<0,05) and a trend towards improvement can be observed at day 40. At day 50 GSE treated flies (purple bars) showed a trend towards improvement which did not reach significance. (B) Compilation of three independent experiments for 40- and 50-day old flies. A significant decrease of cardiac period (measured as the ratio of periods between treated flies and control flies) was observed for GSE at day 50 and PNE at days 40 and 50. Error bars: SEM, *: p<0.05, **: p<0.01, ***: p<0.001. In A) 21<N<56. In B) N=125, 133, 90, for D40 control, GSE and PNE and 126, 102, 120 for D50 control, GSE and PNE respectively.

In a first experiment, we found that treating male flies along lifespan with 3% of DCE or OJE has no significant effect on heart function in aging flies (Fig. 8A and Tab 1). In contrast, treatment with PNE significantly counteracts the increase of the cardiac period in 50-day old flies. At the same age, a trend towards reduction of cardiac period is also seen with GSE treatment but it does not reach significance, which may be due to the limited number of flies analyzed. We performed 2 additional experiments with GSE and PNE on old flies. In all three experiments, the cardiac period of these old flies was reduced compared to untreated flies (Tab 1). Combining all these data showed that, compared to control animals, cardiac period was significantly lowered by both treatments in the oldest flies, while cardiac period of 40-day old flies was significantly lowered only by PNE (Fig. 8B). However, in any case, the rescue of the age-related increase of cardiac period by HEs treatment was only partial when compared to the youngest flies. These findings confirm that some HEs may have positive effects on healthspan on aging individuals.

### GSE and PNE mitigate HD pathology in flies

**Figure 9.**
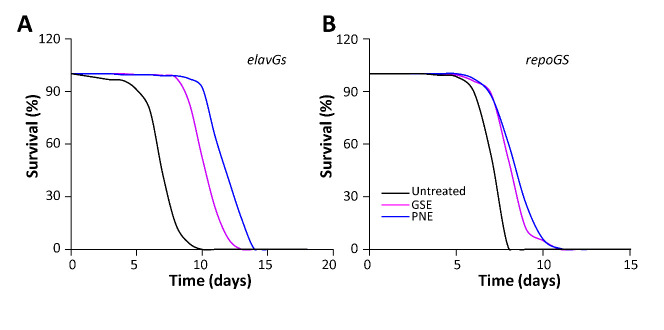
GSE and PNE mitigate HD pathology in neuronal and glial Drosophila models. Flies expressing a pathological Htt-548-128Q protein either in neurons (elavGS>Htt-548-128Q/+ flies, (A) or in glial cells (repoGS>Htt-548-128Q/+ flies, (B) after induction at day 1 with RU486 (black lines) presented reduced lifespan (<10 days) compared to control flies (>40 days, not shown on the graphs). Treatment with GSE (purple lines) or PNE (blue lines) rescued significantly this phenotype (p<0.0001, Logrank test) for both genotypes.

Among a plethora of cellular impairments in Huntington disease, increased oxidative and ER stresses have been reported [33]. Since GSE and PNE protect flies against these stresses in our previous experiment, we asked whether, in similar conditions, these compounds improved HD pathological features. A pathological isoform of human Htt (Htt-548a.a.-128Q) was expressed either in neurons or glial cells in adult flies, using GeneSwitch system. We found that treatment of adult flies with 3% of GSE or PNE provide a significant increase of lifespan to these flies, compared to control flies (Fig. 9).

**Tab 1:** Summary of cardiac function experiments.

Raw data Age	*Compound*	*N*	Set 1 *Mean DT*	*SD DT*	*N*	Set 2 *Mean DT*	*SD DT*	*N*	Set 3 *Mean DT*	*SD DT*
40	Control	41	214.2	9.84	32	253.8	15.45	52	265	7.28
40	GSE	33	224.4	11.39	37	200.9	12.49	63	278.9	7.09
40	PNE	24	198.7	12.47	29	207.3	11.66	37	242.5	8.04
50	Control	43	282.7	27.26	51	311	28.11	32	316.8	15.57
50	GSE	37	228.4	11.61	37	280.4	19.11	28	217.3	7.18
50	PNE	46	212.8	10.62	35	257.9	23.63	39	267.2	10.36
Normalized data			Set 1			Set 2			Set 3	
Normalized data Age	*Compound*	*N*	Set 1 *Mean DT*	*SD DT*	*N*	Set 2 *Mean DT*	*SD DT*	*N*	Set 3 *Mean DT*	*SD DT*
40	Control	41	1.00	0.05	32	1.00	0.06	52	1.00	0.03
40	GSE	33	1.05	0.05	37	0.79	0.05	63	1.05	0.03
40	PNE	24	0.93	0.06	29	0.82	0.05	37	0.92	0.03
50	Control	43	1.00	0.10	51	1.00	0.09	32	1.00	0.05
50	GSE	37	0.81	0.04	37	0.90	0.06	28	0.69	0.02
50	PNE	46	0.75	0.04	35	0.83	0.08	39	0.84	0.03

Upper panel: raw data. The number of flies (N), the mean (mean DT) and the standard error (SD DT) of the intervals between two consecutive heart beats are indicated for the three independent experiments, with the age of the flies and their treatment reported in the first two columns. Lower panel: normalized values compared to control flies.

## DISCUSSION

We have conducted extensive studies on four TCM products (DCE (*Dendrobium candidum* extract), OJE (*Ophiopogon japonicus* root extract), GSE (*Ganoderma sinense* extract) and PNE (*Panax notogingseng* root extract)), investigating with the same extracts: functional stress resistance, longevity of treated animals, improvements of aging hallmarks and ability to rescue Huntington disease.

Previous analysis of these HEs have shown that they contain compounds able to provide protection against oxidative stress [34-38], suggesting that they could be useful as anti-aging agents. However, most of these results have been obtained *in vitro* on different cell types or *in vivo* on pathological conditions and by assessing one oxidative insult. Here, we addressed whether HEs supplementation is able to provide significant *in vivo* protection over a panel of oxidative stresses. A summary of our results is given in Tab. 2. An important result of this study is that protection against oxidative stress varies widely against the four HEs analyzed, both quantitatively and, more importantly, as a function of the type of stress experienced by the flies (see Tab 2). This is reminiscent of the protective responses against specific oxidative stresses that have also been described in genetic studies in yeast and Drosophila [29, 30]. It is thus possible that the TCM compounds we tested activate some genetic pathways mediating oxidative stress specific responses. In this respect, GSE and PNE, that provide protection against the three oxidative stresses assayed, would activate a larger number of protective pathways than DCE or OJE that failed to provide full protection *in vivo*. In a first attempt to identify protective pathways involved in GSE and PNE mediated protection, we took advantage of a viable loss of function (LOF) Foxo mutant. We found that in these flies GSE and PNE are still protective against paraquat induced stress, indicating that the Foxo pathway is not critically involved in the protection mediated by GSE and PNE. In the future, to go further and notably to be able to identify multiple protective pathways working in parallel, will require large scale transcriptional analysis performed in RNAseq experiments. Promoter analysis of differentially expressed genes in flies submitted to stress treatment and supplementation of HEs compared to control flies should help to identify the major regulators of HEs induced protection. Subsequently, LOF and GOF experiments could be used to validate functionally such regulators, as successfully performed in other experimental contexts [25].

Another insult that has been implicated in aging is ER stress. Thus, we tested the ability of TCM HEs to protect the flies against a tunicamycin induced ER stress. Once again, we found significant compound-specific differences that do not necessarily coincide with anti-oxidative properties. For instance, DCE, the less potent compound against oxidative stress, appeared to be the more efficient in the ER stress assay, followed by GSE and PNE. Only two recent papers describe ER stress protection of GSE and PNE in cell studies [39, 40]. Our work extends these findings to *in vivo* protection provided by GSE and PNE in non-pathological conditions and describes for the first time the potency of DCE in counteracting this stress. Altogether, our data point out the need to extend the number of stressors in *in vivo* and *in vitro* studies, to characterize more extensively the properties of TCM HEs and being able to combine these compounds more efficiently on a solid experimental basis.

When we turned to lifespan measurements in HEs-treated flies, we could not detect beneficial effects on medium lifespan, in spite of their significant effects on age-related stress. We thought that this could result from a combination of positive effects, as observed in short term experiments with stresses, and long-term deleterious effects of the compounds. However, we did not detect increases in lifespan when flies were treated with a lower (0.3%) concentration (Supplementary Fig. 4). Similarly, lifespan was unchanged when flies were reared on a poorer diet medium combined with 3% HEs treatment (data not shown). Altogether, these results suggest that the TCM extracts used in this study are unable to extend drosophila lifespan when administered orally. This contrasts with the lifespan increase observed in one experiment by Feng et al. in which *Panax notoginseng* polysaccharides was administered to the nematode *C. elegans* [41]. Differences between Feng’s study and ours could be explained either by the different composition of the extracts of *P. notoginseng* used or, alternatively, by organism specificities. Additional experiments in parallel with the two species may help to solve this issue.

**Tab 2:** Summary of the effect of HEs treatments on stress resistance and longevity.

Compound short name	DCE	OJE	GSE	PNE
Paraquat	+	++	+++	+++
H_2_O_2_	++	0	++	+++
Hyperoxia	0	0	+	+
Tunicamycin	+	0	++	+++
Lifespan	--	0	0	0

+: weak protection (7-12% improvement), ++: medium protection (13-20% improvement), +++: strong protection (>20% improvement), 0: no effect, --: significant decrease (-17%).

One limitation of our study is the relatively limited range of concentration of HEs that we used, as we favored comparison between several compounds over a large set of phenotypes. Thus, we cannot exclude that the effects described in our study could be different at other dosages, for instance if hormetic responses to the stressors are involved. However, like paraquat (Fig. 1), we found that a 3% HEs concentration provide a better protection against H_2_O_2_ compared to 0.5% or 1% concentrations. In addition, lifespan experiment with flies reared on low HEs concentration medium did not provide evidence for median lifespan increase (Supplementary Fig. 4). Therefore, increasing rather than decreasing HEs concentrations, may be more relevant for future investigations.

Disconnection between stress protection and increase in lifespan is not specific to our study [42]. In worms, treatment with Epigallocatechin gallate protects against heat and oxidative stress but does not increase lifespan [43]. Similarly, in humans, there is no evidence that dietary antioxidant supplementation increases lifespan [44]. However, this does not mean that such treatments cannot increase healthspan of individuals. Indeed, consumption of green tea, a EGCG rich beverage, is associated with healthy aging in humans [45]. To investigate whether treatments with the two most efficient TCM compounds (PNE and GSE) could provide some benefits to the flies in term of healthspan, we measured two features that decline with age, spontaneous activity and heart rate, at different time points. We found that both HEs treatments attenuate these age-related declines. Interestingly, we have previously shown that heart-specific overexpression of catalase is able to counteract the heart rate decline in aged flies [25]. Thus, one potential mechanism of the action of PNE and GSE on heart of aging individuals could result from their ability to protect the organism against H_2_O_2_ induced stress, as demonstrated in this study. Previously, PNE and GSE have been shown to have protective properties against several heart diseases (reviewed in [46, 47]) such as myocardial ischemia/reperfusion (I/R) injury or experimentally induced cardiac hypertrophy in mice. Other studies in mice suggest that GSE might improve multiple cardiovascular risk factors, but this was not confirmed on diabetic patients in a recent study [48]. However, our data suggest that both PNE and GSE could have a preventive action on age-related heart dysfunction in normal individuals. As transcriptome analyses of aging hearts have been instrumental to identify new regulators and pathways [25], it would be interesting in the future to investigate whether GSE and PNE are able to change the transcriptomic signature of the aging heart.

Another aspect of aging is the increased incidence of neurodegenerative diseases, as well as the increased penetrance found in their familial forms. Encouraged by the properties of TCM products for protection against oxidative and ER stress in young flies, we investigated whether these compounds may be protective in Drosophila models of HD, the most common polyQ-extended repeat disease in humans. We found that GSE and PNE are able to provide a mild protection in these models. In the future, considering the specific properties of TCM compounds identified in this study, it would be interesting to investigate whether combination of TCM HEs increase protection in HD models. During the course of our study, Liu et al. demonstrated that ginseng treatment delays the onset of a Parkinson-like phenotype in PINK1 depleted flies [49]. Together with our study, this suggests that TCM HEs may be useful on an extended panel of age-related neurodegenerative diseases and should motivate new studies on available Drosophila models before extension to mice models.

Altogether, our study demonstrates that TCM compounds present a diversity of protective characteristics and could be useful to delay some age-related traits and/or the penetrance of an age-related disease. For the first time, using a CaloFly apparatus, we showed that these compounds do not act by modifying the oxidative metabolism in flies *in vivo*. The pathways targeted by these compounds are still elusive and need to be identified. In a first attempt toward this goal, we conclusively demonstrated in this study that the Foxo pathway is not required for oxidative stress protection. We think that, in the next years, systematic transcriptomic studies should enlighten this issue and provide some key answer on the specific action of TCM products.

## Supplementary Materials

The Supplemenantry data can be found online at: www.aginganddisease.org/EN/10.14336/AD.2020.0714-1.


