# Supplementary material for: G. sinense and P. notoginseng Extracts Improve Healthspan of Aging Flies and Provide Protection in A Huntington Disease Model
Source: Aging Dis. 2021 Apr 1;12(2):425–40. doi: 10.14336/AD.2020.0714-1 (PMC7990376; doi:10.14336/AD.2020.0714-1)
Supplement: Supplementary file 1 [file AD-12-2-425-s.pdf]

SUPPLEMENTARY DATA

G. sinense and *P. notoginseng* Extracts Improve Healthspan of Aging Flies and Provide Protection in A Huntington Disease Model

Serafino Teseo[1,2,#], Benjamin Houot[1,#], Kaiye Yang[3,#], Véronique Monnier[1], Guangrong Liu[3,*] Hervé Tricoire[1*]

© 2020. Teseo S et al. Published online at http://www.aginganddisease.org/EN/10.14336/AD.2020.0714-1

SUPPLEMENTARY DATA

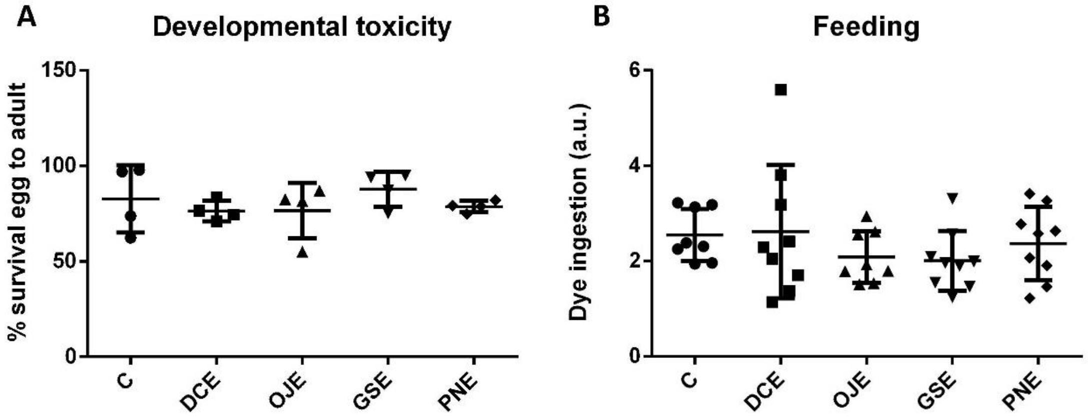

Supplementary Figure 1. HEs do not exhibit significant developmental toxicity and do not modify feeding behavior. A) Developmental survival from eggs to adult flies on medium supplemented by 1% (V/V) of each HE was scored and compared to survival on control (unsupplemented) medium (light gray bar). No statistically significant change in survival were observed. **B**) Feeding behavior was scored in adult flies reared on control medium or medium supplemented by 3% (V/V) of each HE with the described dye absorption method. No statistically significant changes in dye absorption were observed.

© 2020. Teseo S et al. Published online at http://www.aginganddisease.org/EN/10.14336/AD.2020.0714-1

SUPPLEMENTARY DATA

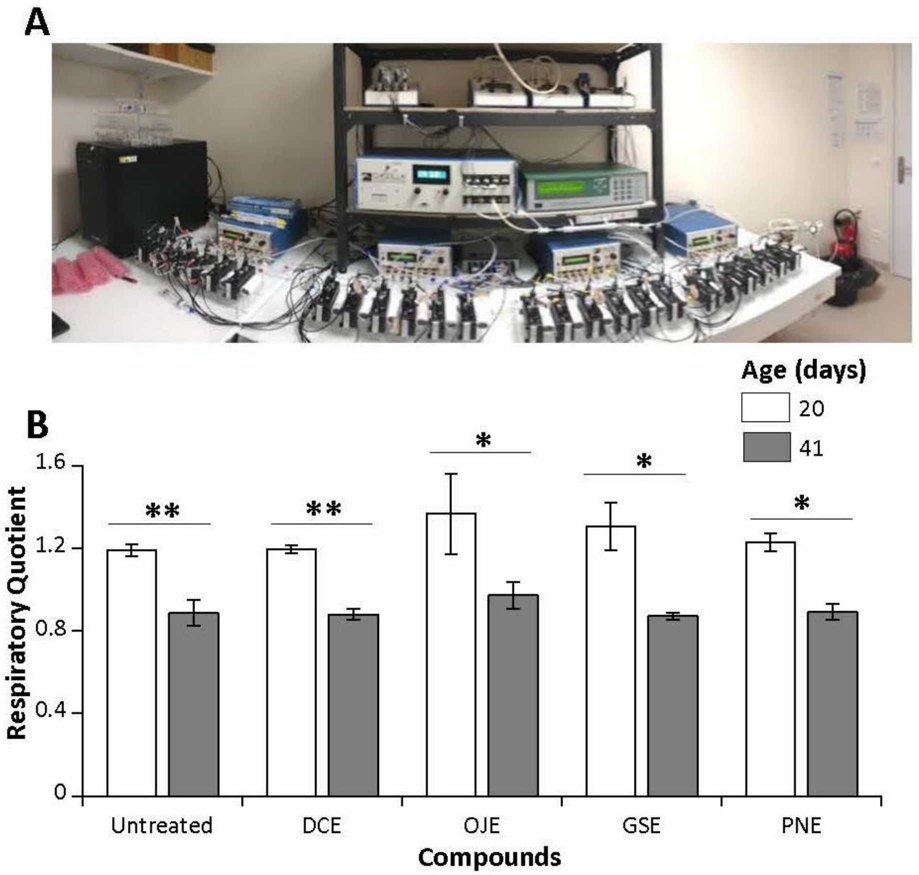

Supplementary Figure 2. Measurement of metabolic activity of flies submitted to HEs treatment. A) The Calofly device with 32 independent chambers used to measure simultaneously O_2 consumption and CO_2 production of individual flies. **B)** The respiratory quotient measured from data of fig. 4 is significantly reduced between 20 day old and 41 day old flies, irrespective of HEs treatment.

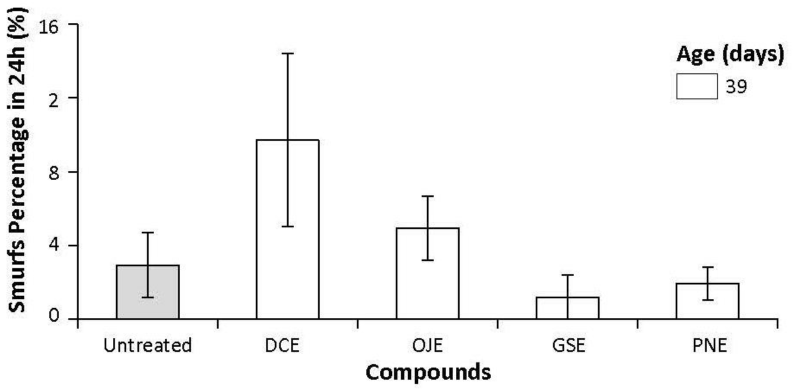

Supplementary Figure 3. Percentage of Smurf flies at day 39. No significant differences were observed between control flies and HEs treated flies ($p>0.05$). Error bars: SEM. 10 tubes of 30 flies were analyzed per condition.

© 2020. Teseo S et al. Published online at http://www.aginganddisease.org/EN/10.14336/AD.2020.0714-1

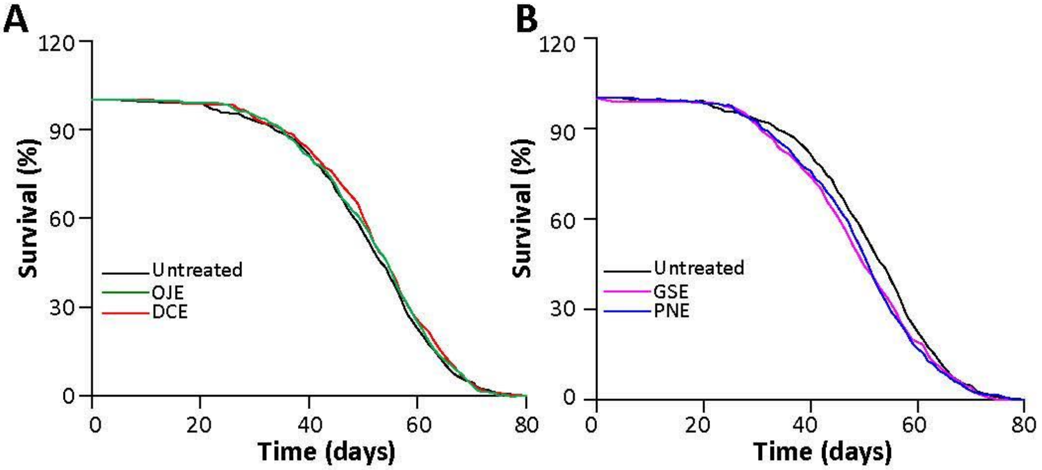

C

Survival (0,3% HEs)					
**Compound**	**Control**	**OJE**	**DCE**	**GSE**	**PNE**
N	296	289	289	302	294
Mean Lifespan	50,0	51,1	50,6	47,8	47,9
**pVal (LogRank)**		3,6E-01	7,2E-01	5,2E-02	5,8E-02

Supplementary Figure 4. Low concentration HEs treatments do not increase fly lifespan. Flies were treated with 0.3% of HEs along their lifespan and survival curves were scored. **A)** DCE and OJE treatment. **B)** PNE and GSE treatment **C)** Summary of the statistical analysis of these curves. In no case the survival curve significantly differs from the control (Logrank analysis, $p>0.05$). A large number of flies ($N_{flies}> 289$ for each condition) were included in this experiment.

© 2020. Teseo S et al. Published online at http://www.aginganddisease.org/EN/10.14336/AD.2020.0714-1
